# Supplementary figures and images for: Hematological and gene co-expression network analyses of high-risk beef cattle defines immunological mechanisms and biological complexes involved in bovine respiratory disease and weight gain
Source: PLoS One. 2022 Nov 3;17(11):e0277033. doi: 10.1371/journal.pone.0277033 (PMC9632787; doi:10.1371/journal.pone.0277033)

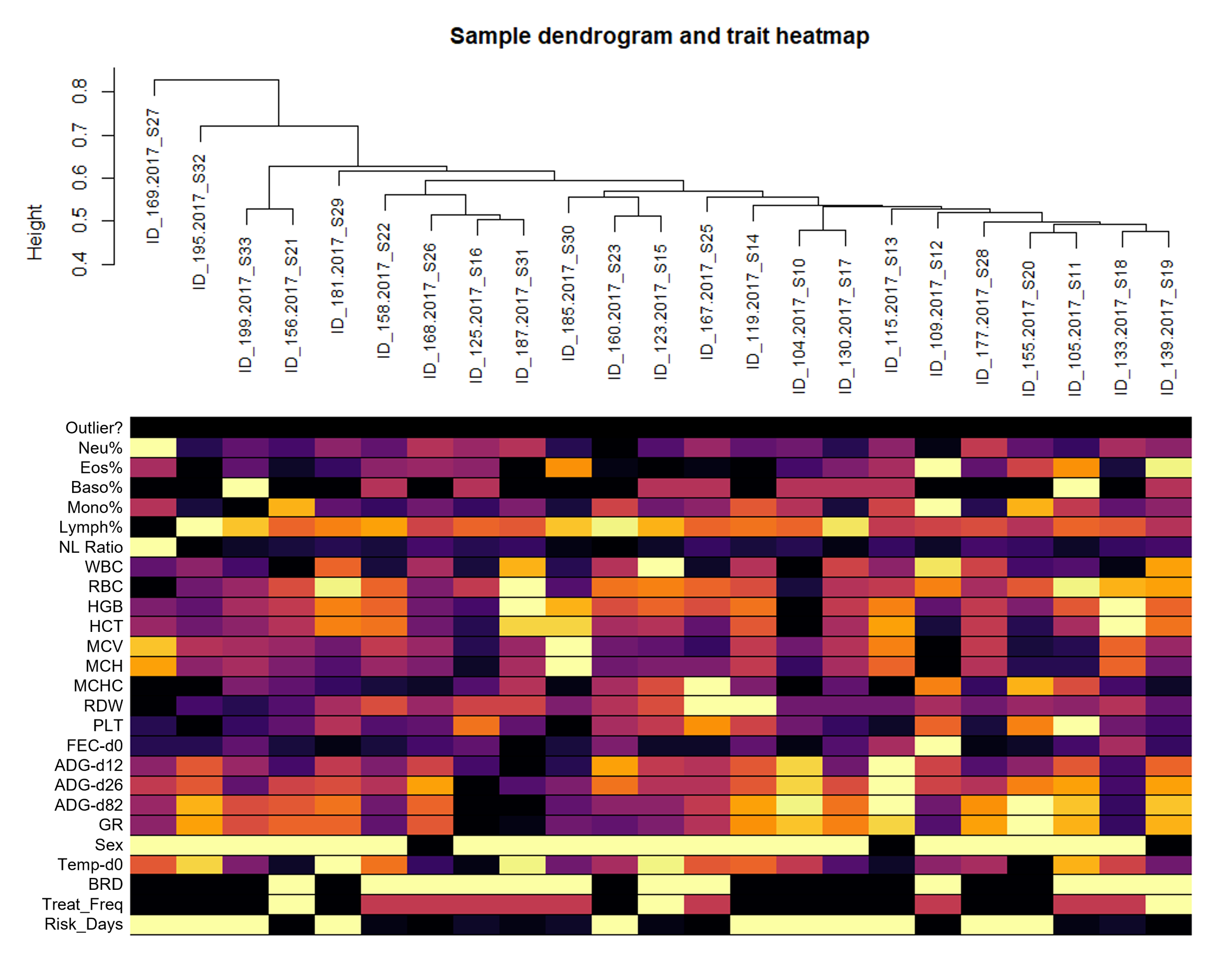

Supplement: S1 Fig — Standardized connectivity was calculated from network adjacency matrices and used to classify potential outliers (Z.k < -5); no animal was identified as an outlier. The remaining rows represent the numerical values of all clinical and hematological traits across each animal. Colors indicate an increase (yellow/white) or decreased (purple/black) value for each trait; Sex and BRD are both represented as a value of 1 for bulls and Yes, and 0 for steers and No, respectively. (TIF) [file pone.0277033.s007.tif]

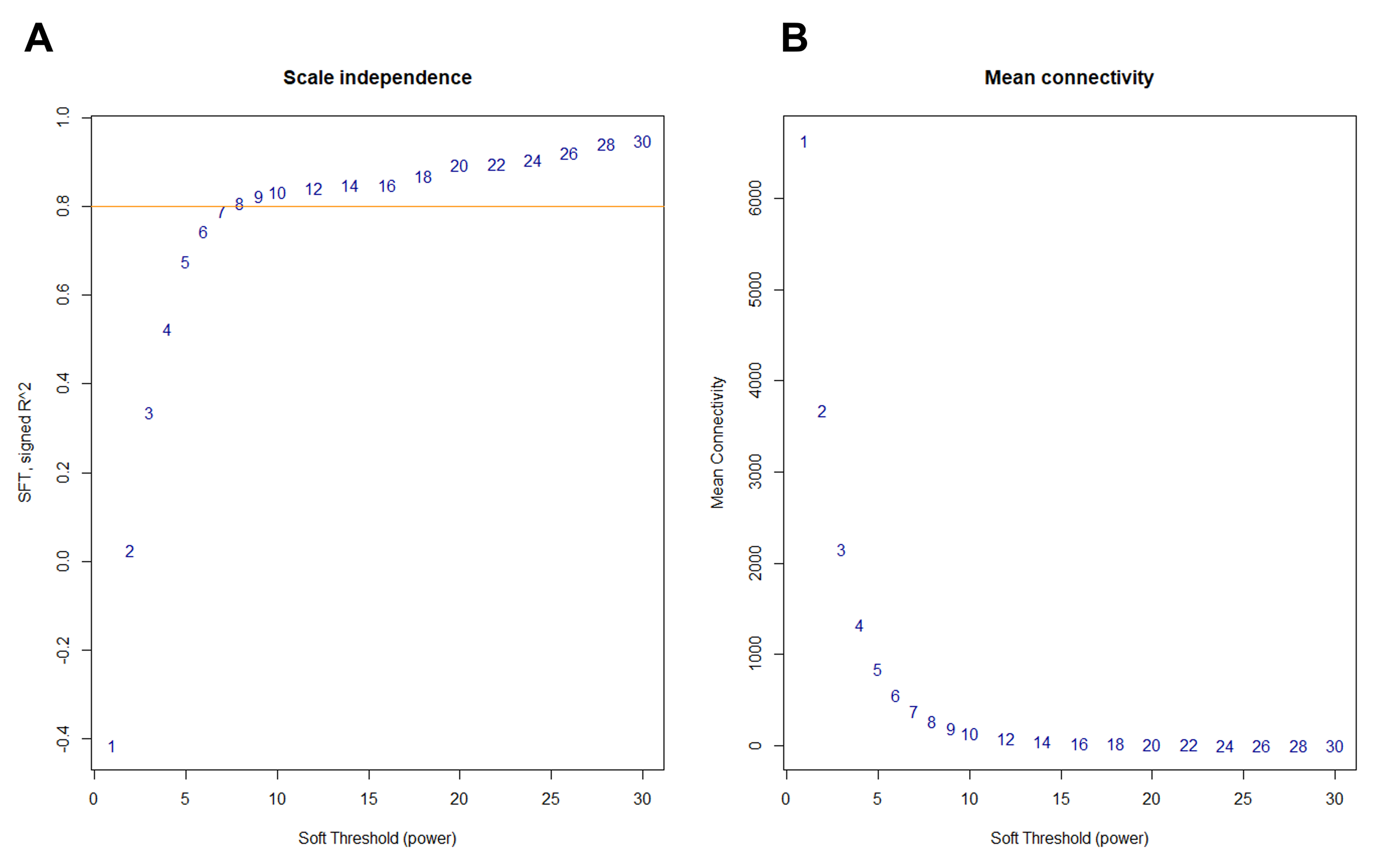

Supplement: S2 Fig — A) SFT index R2 (y-axis) at increasing soft threshold powers (β; x-axis). The value β = 8 was selected, seen where the saturation curve is above 0.8 (orange horizontal line). B) Increasing soft threshold powers (β; x-axis) with respect to decreasing mean connectivity (y-axis). The goal of selecting a value β is to maximize scale independence (i.e., suppress low correlations) while simultaneously minimizing loss in mean connectivity. (TIF) [file pone.0277033.s008.tif]
