# Supplementary material for: Hematological and gene co-expression network analyses of high-risk beef cattle defines immunological mechanisms and biological complexes involved in bovine respiratory disease and weight gain
Source: PLoS One. 2022 Nov 3;17(11):e0277033. doi: 10.1371/journal.pone.0277033 (PMC9632787; doi:10.1371/journal.pone.0277033)
Supplement: Attachment — Submitted filename: Final decision from PCOMPBIOL-D-22-00235.docx [file pone.0277033.s009.docx]

Dear Dr. Scott,

Thank you very much for submitting your manuscript "Hematological and gene co-expression network analyses of high-risk beef cattle defines immunological mechanisms and biological complexes involved in bovine respiratory disease and weight gain" (PCOMPBIOL-D-22-00235) for consideration at PLOS Computational Biology. As with all papers, your manuscript was reviewed by members of the editorial board. Based on our initial assessment, we regret that we will not be pursuing this manuscript for publication at PLOS Computational Biology.

The work examines previously published RNA-seq data from cattle, uses WGCNA to find co-expression modules and relates these modules to clinical and hematological characteristics of the animals. The analysis led to specific hypotheses about molecular pathways and hub genes possibly related to Bovine Respiratory Disease (BRD) development. We feel that the appeal of the work is likely to be in the application of existing computational methodology to a biological problem, rather than the development of a novel method or pipeline.

We are sorry that we cannot be more positive on this occasion. We very much appreciate your wish to present your work in one of PLOS's Open Access publications. Thank you for your support, and we hope that you will consider PLOS Computational Biology for other submissions in the future.

Sincerely,

Saurabh Sinha

Guest Editor

PLOS Computational Biology

Ilya Ioshikhes

Deputy Editor

PLOS Computational Biology

__________________________________________________

In compliance with data protection regulations, you may request that we remove your personal registration details at any time. (Use the following URL: https://www.editorialmanager.com/pcompbiol/login.asp?a=r). Please contact the publication office if you have any questions.
