# Supplementary material for: Hematological and gene co-expression network analyses of high-risk beef cattle defines immunological mechanisms and biological complexes involved in bovine respiratory disease and weight gain
Source: PLoS One. 2022 Nov 3;17(11):e0277033. doi: 10.1371/journal.pone.0277033 (PMC9632787; doi:10.1371/journal.pone.0277033)
Supplement: Attachment — Submitted filename: Response to Reviewers.docx [file pone.0277033.s010.docx]

Dear Dr. Jianhua Ruan,

Thank you for your letter containing the publishing decision and review of our manuscript, "Hematological and gene co-expression network analyses of high-risk beef cattle defines immunological mechanisms and biological complexes involved in bovine respiratory disease and weight gain". Additionally, we thank the reviewers for their time and constructive feedback in efforts to improve the clarity and quality of our manuscript.

Below, we have provided and addressed all comments (in blue letters) and have adopted changes within the manuscript; all adopted changes, including line numbers, are conveyed through Microsoft Word Track Changes in the revised manuscript document. Again, we thank you for your time and hope our revised manuscript is now acceptable for publication.

Sincerely,

On behalf of all authors

Matthew A. Scott DVM, PhD

**Editor Comments:**

Journal requirements:

When submitting your revision, we need you to address these additional requirements.

1. Please ensure that your manuscript meets PLOS ONE's style requirements, including those for file naming. The PLOS ONE style templates can be found at https://journals.plos.org/plosone/s/file?id=wjVg/PLOSOne_formatting_sample_main_body.pdf and https://journals.plos.org/plosone/s/file?id=ba62/PLOSOne_formatting_sample_title_authors_affiliations.pdf".

- The revised manuscript and title page have been edited to fit the PLOS ONE style requirements.

2. We note that the grant information you provided in the ‘Funding Information’ and ‘Financial Disclosure’ sections do not match. When you resubmit, please ensure that you provide the correct grant numbers for the awards you received for your study in the ‘Funding Information’ section.

- We have ensured that the correct grant number is referred to in the Funding Information.

3. Thank you for stating the following in the Acknowledgments Section of your manuscript:

“This work was supported by the Animal Health and Disease Program [Grant no. 2020-67016-31469] from the USDA National Institute of Food and Agriculture. The authors thank William Crosby, Kirsten Midkiff, Alexis Thompson, Joseph Gerlach, Merrilee Thoresen, and the supporting staff at the Mississippi State University College of Veterinary Medicine Clinical Pathology Laboratory for their technical assistance and insights throughout this study. The authors would also thank students and staff of the Mississippi Agricultural and Forestry Experiment Station (MAFES) and Mississippi State University Animal and Diary Science Department for their assistance in animal care and sample collection.” We note that you have provided funding information that is not currently declared in your Funding Statement. However, funding information should not appear in the Acknowledgments section or other areas of your manuscript. We will only publish funding information present in the Funding Statement section of the online submission form. Please remove any funding-related text from the manuscript and let us know how you would like to update your Funding Statement. Currently, your Funding Statement reads as follows: “The funders had no role in study design, data collection and analysis, decision to publish, or preparation of the manuscript.” Please include your amended statements within your cover letter; we will change the online submission form on your behalf.

- We have removed the funding information from the Acknowledgements section of the manuscript. We have included the grant number reference (USDA National Institute of Food and Agriculture Animal Health and Disease Program Grant no. 2020-67016-31469) in the Funding Statement. We would like to include “This work was supported by the Animal Health and Disease Program [Grant no. 2020-67016-31469] from the USDA National Institute of Food and Agriculture. The funders had no role in study design, data collection and analysis, decision to publish, or preparation of the manuscript.”

4. We note that you have stated that you will provide repository information for your data at acceptance. Should your manuscript be accepted for publication, we will hold it until you provide the relevant accession numbers or DOIs necessary to access your data. If you wish to make changes to your Data Availability statement, please describe these changes in your cover letter and we will update your Data Availability statement to reflect the information you provide.

- The GEO submission # GSE161396 is currently publicly available. We will make the repository submissions listed in the Data Availability section (GitHub, Zenodo) publicly available upon publication acceptance.

**Reviewer Comments:**

Reviewer #1

1. The manuscript cited a previous publication. However, the description of animal population was still not adequate. It would be more informative to briefly describe how animals were selected for the study in 2017 and in 2019. Was there any information about animal breed composition or major breeds? Were the animals in similar ages at arrival? If not, was the age difference significant between the BRD and Healthy animal groups? Were the cattle related by pedigree, i.e., possible family structures within the BRD or health groups? If the animal information was not available, please indicate and discuss possible impacts on the results.

- Information regarding the cattle populations is described in lines 131-138. Weights are found in Supplementary Table S1 and are proportional to one-another, and ages (not recorded) were estimated to be similar upon facility arrival. Regarding the reviewer’s comments on animal pedigree, these animals were purchased through a commercial order-buyer, through an auction system, and are considered of commercial *Bos taurus* breed. Although the physical appearance of the cattle suggests that they are likely the result of crossbreeding between cattle of certain breeds (e.g., Angus, Hereford, Simmental, and/or Charolais), the exact breeds represented are not known. As their genetic makeup and traits are unmeasured, this information cannot be accounted for in a statistical model. This heterogeny is common in beef cattle production systems, as the studies in which these cattle were enrolled into was focused on simulating commercial management practices routine of the US beef industry.

2. The manuscript reports clinical data for these cattle in Supplemental Table S1. The Table has 23 cattle, which seems to be animals of 2017. How about the clinical data of cattle of 2019?

- The clinical data from cattle in 2019 is described in our previous manuscript (citation 20) and now in lines 131-138. It has been added to Supplemental Table S1.

3. Supplemental Table S1 listed data of WT-d0. However, the comparison of WT-d0 was not included in Table 1. Were the average body weights at arrival significantly different between the BRD and Healthy animals? If yes, was the body weight difference associated with BRD development?

- We did not include the at arrival weight (WT-d0) as an analysis variable because there was no significant difference between the Healthy (mean: 477.0, s.d.:24.8) and BRD (mean: 475.3, s.d.:26.9) cohorts.

4. Line 183, please describe and define the elements in the equation.

- Lines 190-192 have been added to define the elements of the equation used to determine outliers.

5. Pearson correlations were calculated to assess the relationship between modules and hematological clinical traits. However, some trait values did not follow a normal distribution. Would this bias the estimation of the correlation coefficients? Please discuss.

- The majority of variables tested for module correlations were normally distributed. The nine (of the 26 total) non-parametric testing variables’ distributions are plotted in the internal document (titled “QQPlot”). Visual assessment demonstrates relative linearity with six of the nine non-parametric variables (based on Shapiro-Wilk testing for normality (line 282)), with few high margin data points: Neu%, Lymph%, NL Ratio, MCHC, RDW, and FEC-d0. The non-parametric nature of Eos%, Baso%, and Sex is perceived to be due to data sparsity and relative rarity of the expected cell counts attributed to eosinophils and basophils in cattle (DOI: 10.1016/j.cvfa.2007.07.002; see internal reference values on Table 1). We elected to utilize Pearson correlation models as they can measure discrete and continuous datasets without need for transformation, and preserve linearity from the raw data structure when assessing these variables with gene co-expression modules. Additionally, calculated Pearson correlations from WGCNA can better handle datasets with missing or censored data and is highly computationally efficient (DOI: 10.18637/jss.v046.i11). Overall, the primary findings of this study, related to BRD development and severity, erythrocytes, Th2-type immunity, were not biased by these estimations.

6. The RNAseq data were analyzed previously using differential gene expression analyses, and differentially expressed genes (DEGs) related to BRD were identified. Please discuss more on how the identified DEGs in the previous study are related to results from the current study including the hub genes detected.

- We have added lines 617-623 to the discussion to provide more detail regarding the previously identified DEGs in relation to BRD development stemming from hub gene analysis.

7. The image quality of Figure 2-5 should be improved.

- All original high-resolution images have been process and converted by the Picture Analysis and Conversion Engine (PACE; <https://pacev2.apexcovantage.com/>), per submission instructions provided by PLOS ONE (<https://journals.plos.org/plosone/s/figures>).

Reviewer #2

The authors investigated the co-expression network analysis in bovine respiratory disease and weight gain traits. This subject is within the scope of the journal and the results would provide insight and understanding about bovine respiratory disease and weight gain. Although the manuscript has merits in many aspects, there are several points need to be clarified, I suggest a minor revision.

1.Collectively, there are a number of grammatical typos and some spelling mistakes. Please check the manuscript carefully and revise the manuscript thoroughly.

- The manuscript has been evaluated and corrected for any potential grammatical or spelling errors. Color coding terms (such as steelblue) are identifiers provided by WGCNA and word processing programs will depict these terms as spelling mistakes.

2.Please improve the quality of the figures.

- All original high-resolution images have been process and converted by the Picture Analysis and Conversion Engine (PACE; <https://pacev2.apexcovantage.com/>), per submission instructions provided by PLOS ONE (<https://journals.plos.org/plosone/s/figures>).

3. I need to check the WGCNA code that you used for analysis. Please put the WGCNA code in the supplementary file.

- The code and data used for this manuscript for all analyses is stored in a GitHub repository, which will be made publicly available upon manuscript publication (please see Data Availability statement). For verification of R coding, an R script and text file (both titled “2022_BRD_WGCNA”) have been added as internal documents for the reviewer.
