# Supplementary material for: Hematological and gene co-expression network analyses of high-risk beef cattle defines immunological mechanisms and biological complexes involved in bovine respiratory disease and weight gain
Source: PLoS One. 2022 Nov 3;17(11):e0277033. doi: 10.1371/journal.pone.0277033 (PMC9632787; doi:10.1371/journal.pone.0277033)
Supplement: Attachment — Submitted filename: Response to Reviewers.docx [file pone.0277033.s011.docx]

Dear Dr. Jianhua Ruan,

Thank you for your letter regarding the comments to our revised manuscript, "Hematological and gene co-expression network analyses of high-risk beef cattle defines immunological mechanisms and biological complexes involved in bovine respiratory disease and weight gain". Once again, we thank the editorial team and reviewers for their time and constructive feedback to our manuscript.

Below, we have provided and addressed all comments (in blue letters) and have adopted changes within the manuscript; all adopted changes, including line numbers, are conveyed through Microsoft Word Track Changes in the revised manuscript document. Again, we thank you for your time and hope our revised manuscript is now acceptable for publication.

Sincerely,

On behalf of all authors

Matthew A. Scott DVM, PhD

**Journal Requirements:**

Please review your reference list to ensure that it is complete and correct. If you have cited papers that have been retracted, please include the rationale for doing so in the manuscript text, or remove these references and replace them with relevant current references. Any changes to the reference list should be mentioned in the rebuttal letter that accompanies your revised manuscript. If you need to cite a retracted article, indicate the article’s retracted status in the References list and also include a citation and full reference for the retraction notice.

Two additional citations (#66 and #67) have been added to the manuscript in support of the added discussion suggested by Reviewer #1. Additionally, citation #41 has been updated, as the aforementioned preprint was accepted and published and the changes have addressed this change. All other changes to the reference list were of numerical order (after the addition of citations #66 and #67) and grammatical use. None of the articles referenced have been retracted to our knowledge.

**Reviewer Comments:**

Reviewer #1:

The authors have responded to the reviewer’s comments and have made some revisions. However, it is suggested that the authors incorporate the responses in the manuscript to improve clarity. For example, “ages (not recorded) were estimated to be similar upon facility arrival”, “We did not include the at arrival weight (WT-d0) as an analysis variable because there was no significant difference between the Healthy (mean: 477.0, s.d.:24.8) and BRD (mean: 475.3, s.d.:26.9) cohorts”.

We had added lines 146-147, 303-305, and 548-557, including citations 66 and 67, to incorporate our previous responses to improve clarity of the manuscript.

Reviewer #2:

I have gone through this manuscript and the response letter. I recommended this manuscript for publication in this format.

We greatly appreciate the reviewer’s previous suggestions and current support for publication.
